# A Rapid Murine Coma and Behavior Scale for Quantitative Assessment of Murine Cerebral Malaria

**DOI:** 10.1371/journal.pone.0013124

**Published:** 2010-10-01

**Authors:** Ryan W. Carroll, Mark S. Wainwright, Kwang-Youn Kim, Trilokesh Kidambi, Noé D. Gómez, Terrie Taylor, Kasturi Haldar

**Affiliations:** 1 Department of Pediatrics, Feinberg School of Medicine, Northwestern University, Chicago, Illinois, United States of America; 2 Department of Preventive Medicine, Feinberg School of Medicine, Northwestern University, Chicago, Illinois, United States of America; 3 Department of Pathology, Feinberg School of Medicine, Northwestern University, Chicago, Illinois, United States of America; 4 Center for Rare and Neglected Diseases, University of Notre Dame, South Bend, Indiana, United States of America; 5 Department of Internal Medicine, College of Osteopathic Medicine, Michigan State University, East Lansing, Michigan, United States of America; New York University School of Medicine, United States of America

## Abstract

**Background:**

Cerebral malaria (CM) is a neurological syndrome that includes coma and seizures following malaria parasite infection. The pathophysiology is not fully understood and cannot be accounted for by infection alone: patients still succumb to CM, even if the underlying parasite infection has resolved. To that effect, there is no known adjuvant therapy for CM. Current murine CM (MCM) models do not allow for rapid clinical identification of affected animals following infection. An animal model that more closely mimics the clinical features of human CM would be helpful in elucidating potential mechanisms of disease pathogenesis and evaluating new adjuvant therapies.

**Methodology/Principal Findings:**

A quantitative, rapid murine coma and behavior scale (RMCBS) comprised of 10 parameters was developed to assess MCM manifested in C57BL/6 mice infected with *Plasmodium berghei* ANKA (PbA). Using this method a single mouse can be completely assessed within 3 minutes. The RMCBS enables the operator to follow the evolution of the clinical syndrome, validated here by correlations with intracerebral hemorrhages. It provides a tool by which subjects can be identified as symptomatic prior to the initiation of trial treatment.

**Conclusions/Significance:**

Since the RMCBS enables an operator to rapidly follow the course of disease, label a subject as affected or not, and correlate the level of illness with neuropathologic injury, it can ultimately be used to guide the initiation of treatment after the onset of cerebral disease (thus emulating the situation in the field). The RMCBS is a tool by which an adjuvant therapy can be objectively assessed.

## Introduction

Malaria kills 1–2 million people per year, mostly children under the age of 5 in sub-Saharan Africa [1], [2]. The protozoan parasite *Plasmodium falciparum* is the most common cause of malaria in the world, and by far, the most debilitating and deadly phenotype of malaria is the neurological syndrome cerebral malaria (CM) [3]. At the point of diagnosis, the decline in health is rapid and 10%–20% of treated patients die, often within 24–72 hours after the onset of illness, and 10–15% of survivors are left with neurological sequelae [4], [5]. The pathophysiology of CM is not fully understood, and at present there is no known effective adjuvant therapy.

CM is diagnosed clinically using the criteria set forth by the World Health Organization (WHO): (1) an encephalopathic state with a Glasgow Coma Score (GCS) of 11 out of 15, or less for adults, or a Blantyre Coma Scale (BCS) of 2 out of 5, or less, for children; (2) verified *Plasmodium* parasitemia; (3) exclusion of potential confounding factors such as hypoglycemia, on-going seizures, and/or other CNS infections [6]. Human CM has been described to involve multiple types of CNS dysfunction, including encephalopathy, posturing, seizures, and coma [6], [7].

A well-described mouse model involves a CM-susceptible strain (C57BL/6) infected with the rodent malaria parasite, *Plasmodium berghei* ANKA (PbA) [8]. A proportion of infected subjects develop neurological symptoms and rapidly progress to death within 5 to 10 days of infection, in the face of relatively low parasitemia. In principle, the MCM can be used to study the mechanisms of CM, and to identify and validate new therapeutic targets using clinically relevant disease endpoints. The use of the MCM models has been limited in part by the lack of a rapid scoring system used to objectively assess functional neurologic impairment.

In light of the rapid decline in health in the MCM subjects, and the need for a model to emulate the human CM scenario, we have developed a rapid murine coma and behavior scale (RMCBS). This score utilizes 10 parameters and can be used to assess a subject mouse under 3 minutes, thus allowing a large number of mice to be studied in one experiment, providing the level of throughput and power needed to determine medication efficacy in adjuvant therapy trials. The level of illness, as objectively determined by the RMCBS, was corroborated by inter-operator validation, and was significantly correlated with intracerebral pathology. The RMCBS can be used to objectively label a mouse as affected, or not, providing a starting point for adjuvant therapy trials. This assessment method more closely emulates the situation in the field and attempts to bring the animal model closer to the human disease.

## Results

### Establishment of the RMCBS and its utilization to measure murine cerebral malaria induced by *P. berghei* ANKA infection

The RMCBS consists of ten (10) parameters (see Table 1, and Supporting Information Video S1/Video S2), each scored from 0 as the lowest, to 2 as the highest, with a maximum possible score of 20. We utilized parameters similar to components of the SHIRPA score [9], [10], using only those that reflect real-time CNS function, and those that could be assessed within seconds. The final product is the RMCBS, a tool by which each mouse could be assessed in three minutes, or less, in two steps of 90 seconds each, as described below.

**Table 1:** The Rapid Murine Coma and Behavior Scale (RMCBS).

Label	Score	Description
Coordination		
Gait	(0–2)	(none – ataxic – normal)
Balance	(0–2)	(no body extension – extends front feet on wall – entire body lift)
Exploratory Behavior		
Motor Performance	(0–2)	(none – 2–3 corners explored in 90 seconds – explores 4 corners in 15 seconds)
Strength and Tone		
Body Position	(0–2)	(on side – hunched – full extension)
Limb Strength	(0–2)	(hypotonic, no grasp – weak pull-back[front paw grasp only] – strong pull-back[active pull away, jerk away])
Reflexes and Self-Preservation		
Touch Escape	(0–2)	(none – unilateral – instant and bilateral; in 3 attempts)
Pinna Reflex	(0–2)	(none – unilateral – instant and bilateral; in 3 attempts)
Toe Pinch	(0–2)	(none – unilateral – instant and bilateral; in 3 attempts)
Aggression	(0–2)	(none – bite attempt with tail cut – bite attempt prior to tail cut, in 5 seconds)
Hygiene-Related Behavior		
Grooming	(0–2)	(ruffled, with swaths of hair out of place – dusty/piloerection – normal/clean with sheen)

The RMCBS consists of 10 parameters, and each parameter is scored 0 to 2, with a 0 score correlating with the lowest function and a 2 score, the highest. An animal can achieve an accumulative score of 0 to 20.

In the initial 90 seconds, mice were assessed for hygiene-related behavior, gait, body position, exploratory behavior, and balance (Table 1). Thus, a mouse was scored as healthy in the presence of: (i) smooth, preened hair with sheen (ii) a steady gait (iii) full extension of its body as it sat and walked (iv) explored all four corners of the grid box in under 15 seconds and (v) reached the top of the grid box and pulled itself to the edge, lifting its hind legs off the cage floor. In contrast, an ill mouse respectively developed (i) piloerection, and later complete disarray of its hair (ii) ataxia, or no gait (iii) remained in a crouched position, even while walking, and later lay on its side (iv) explored only three, or fewer corners in 90 seconds, and later did not move from the original corner and (v) placed only its forelegs on the cage edge and later ceased to raise the forelegs altogether.

In the next 90 seconds, mice were assessed for reflexes, limb strength, and self-preservation behaviors (Table 1). A healthy mouse (i) jumped away from the stimulus of a touch to its flank, often on the first offense, and certainly within three attempts made on each side (ii) flicked its ear when the pinna was touched (iii) grasped a 3-mm diameter rod and pulled away (iv) reflexively pulled its hind leg away from a toe pinch while suspended with the front paws grasping the rod and (v) bit at the retention tube and restraining ring when secured for the purposes of tail blood sampling. An ill mouse would respectively demonstrate (i, ii, iv, and v) fewer to no reflexive movements with stimulation, and (iii) would not pull away when suspended, eventually refusing to grasp the 3-mm diameter rod.

To address operator bias, the score was independently validated via video (Table 2). An initial operator (Operator 1: Op 1) scored two symptomatic mice and one asymptomatic control mouse, using the RMCBS, while videotaping the procedure. A second operator (Operator 2: Op 2), blinded to the score and category of the subject (control versus experimental), viewed the video and provided a score. Grooming was the only parameter difficult to capture on film and was not included in this validation; therefore, the highest achievable RMCBS total score was 18. The final scores, when compared between Op 1 and Op 2 resulted in a Krippendorff's alpha of 0.86.

**Table 2:** Comparison of RMCBS scores between two operators.

Label	Score	Mouse 1		Mouse 2		Mouse 3	
		Op 1	Op 2	Op 1	Op 2	Op 1	Op 2
Coordination							
Gait	(0–2)	1	1	2	2	2	2
Balance	(0–2)	0	0	0	0	2	2
Exploratory Behavior							
Motor Performance	(0–2)	0	0	1	2	2	2
Strength and Tone							
Body Position	(0–2)	1	1	1	1	2	2
Limb Strength	(0–2)	1	1	2	2	2	2
Reflexes and Self-Preservation							
Touch Escape	(0–2)	1	0	1	1	2	2
Pinna Reflex	(0–2)	0	0	2	2	2	2
Toe Pinch	(0–2)	0	2	1	2	2	2
Aggression	(0–2)	0	0	2	2	2	2
Hygiene-Related Behavior							
Grooming	(0–2)	1	N/A	1	N/A	2	N/A
Total with grooming	(0–20)	5	N/A	13	N/A	20	N/A
Total without grooming	(0–18)	4	5	12	14	18	18

Symptomatic mice (severe and mild, Mouse 1 and 2, respectively) and a non-infected asymptomatic mouse (Mouse 3) were assessed and videotaped. Operator 1 (Op 1) assessed in real time, while Operator 2 (Op 2) assessed via video footage. Krippendorff's alpha of 0.86.

**Figure 1:**
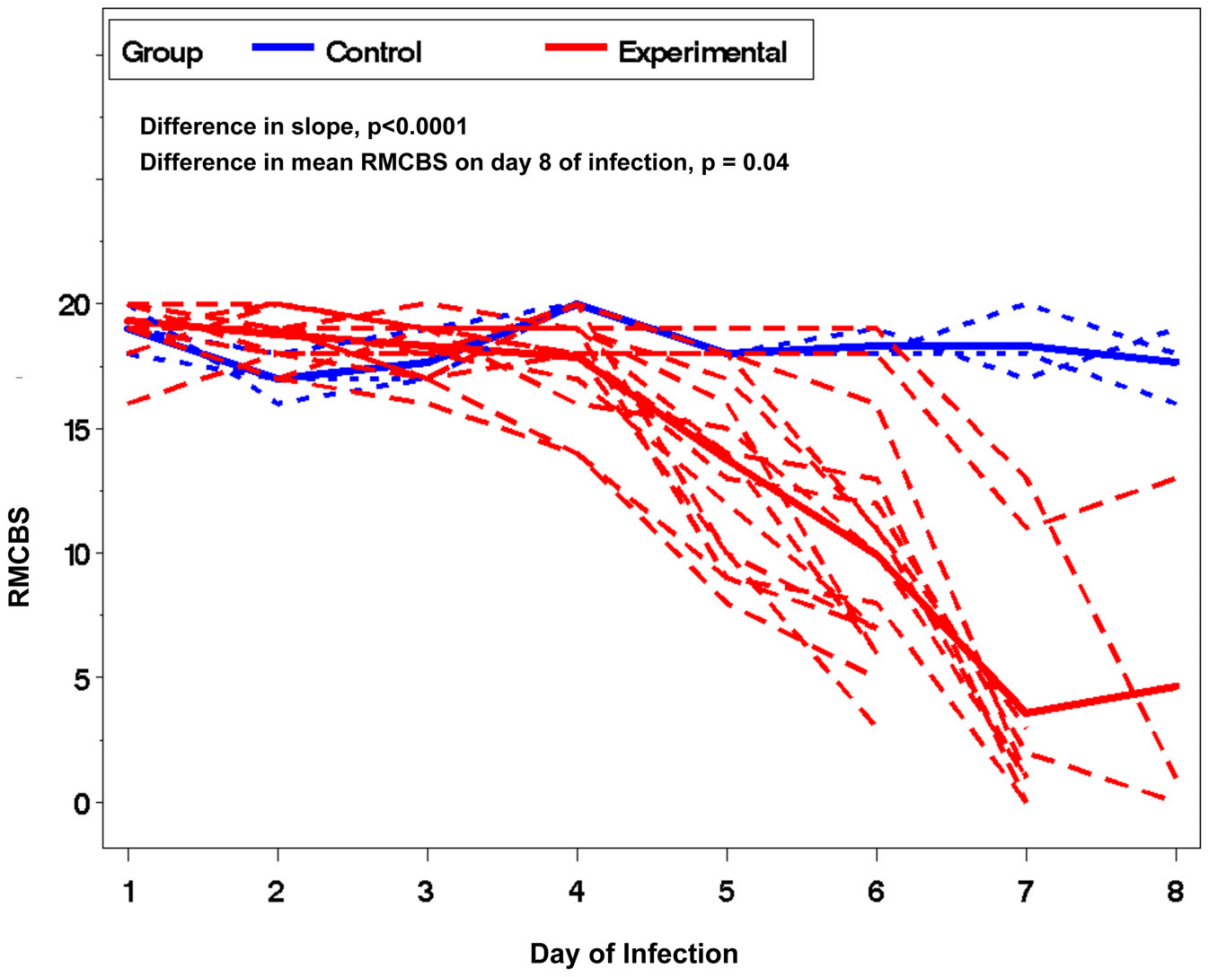
Mean RMCBS and day of infection curves. These data represent the typical trajectory of disease in the MCM model, as highlighted by the RMCBS. Lightly colored lines represent individual subjects, and the two darker lines represent the mean scores from the experimental mice (red dashed line, n = 16) and the control mice (blue dotted line, n = 3). Comparing the mean RMCBS curves was significant, p<0.0001, and the *t*-test to assess the difference in RMCBS at day 8 of infection was also significant, p = 0.04.

To demonstrate the time course of MCM using the RMCBS, we daily assessed experimental mice infected with PbA (n = 16) and non-infected control (n = 3) (Figure 1). The representative number of experimental mice, per day of infection, during the rapid decline in RMCBS were as follows: Day 5, n = 16; Day 6, n = 16; Day 7, n = 8 (50% mortality from the previous day); Day 8, n = 3 (63% mortality from the previous day). Note that mice with scores between 12 and 15 developed a rapid decline in score, and ultimately became moribund within 24 hours.

As shown in Figure 1, the RMCBS score shows a declining linear and quadratic trend for the control and experimental group, respectively. In order to accommodate the different trends in the two groups, we fit a regression with both linear and quadratic terms, resulting in the following models (for mouse i on day j):
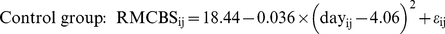


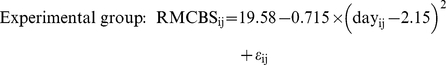
These equations render a difference in the slopes of the two groups (p = 0.0001), and t-test evaluation reveals a significant difference in the mean RMCBS scores on the final day of the experiment (day 8; remaining experimental mice, n = 3, mean RMCBS 4.67±7.23 SD versus control, n = 3, mean RMCBS 17.67±1.53 SD, p = 0.04).

**Figure 2:**
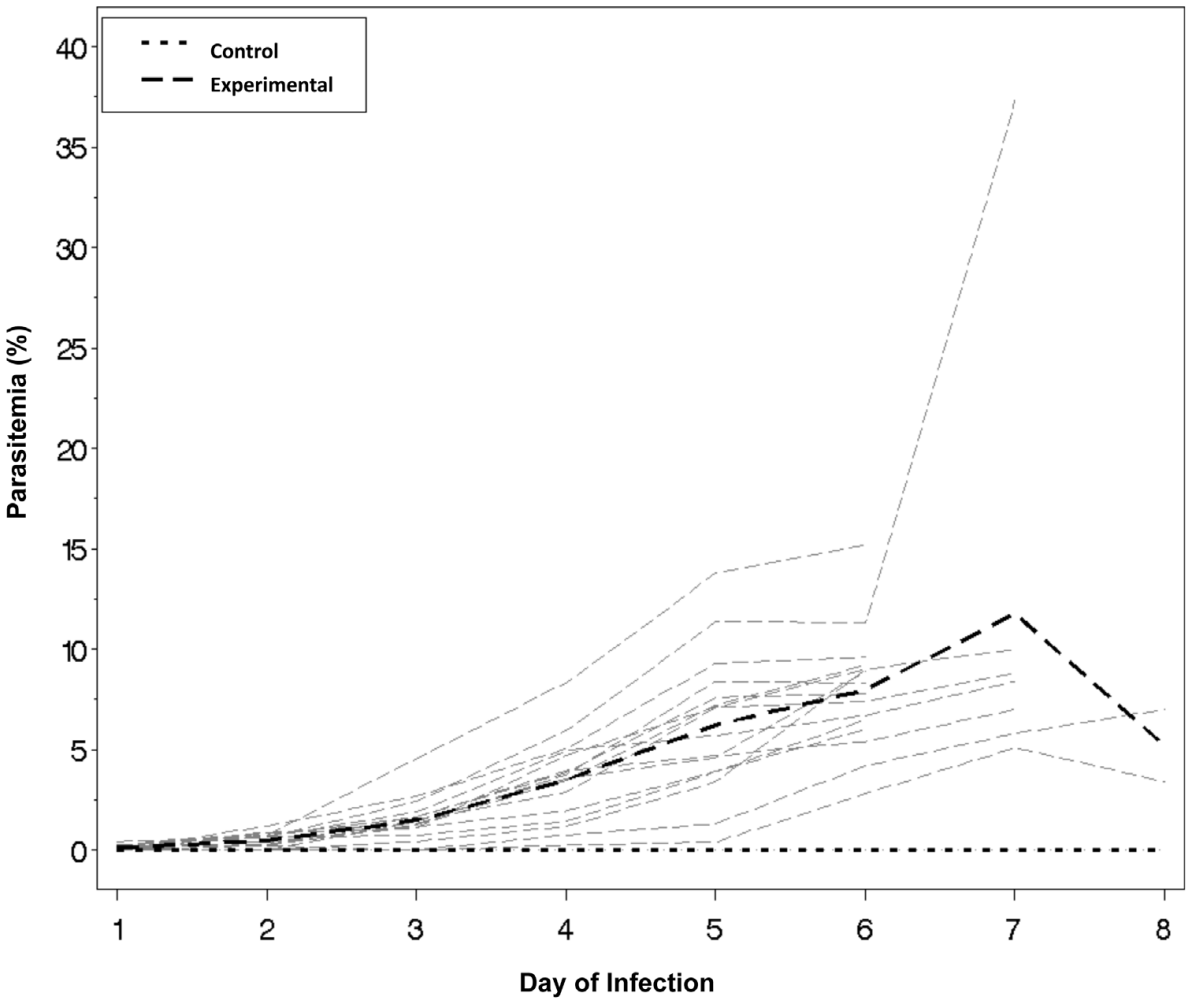
Parasitemia levels in experimental and control mice. Mice inoculated with PbA, as well as non-inoculated controls, were followed daily for parasitemia levels. Mean parasitemia on day 5 was 3.5%, with a peak of 11.8% on day 8 (including an outlier of 37%). Light gray lines represent individual subjects, and the two darker lines represent the mean levels from the experimental mice (dark dashed line, n = 16) and the control mice (dark dotted line, n = 3).

The mice became symptomatic despite low parasitemia levels, with a mean level of 3.5% on day 5 of infection and a peak mean level of 11.8% on day 8 of infection (range of 5.1%–8.8% on day 8 of infection, with an outlier of 37.3%) (Figure 2).

**Figure 3:**
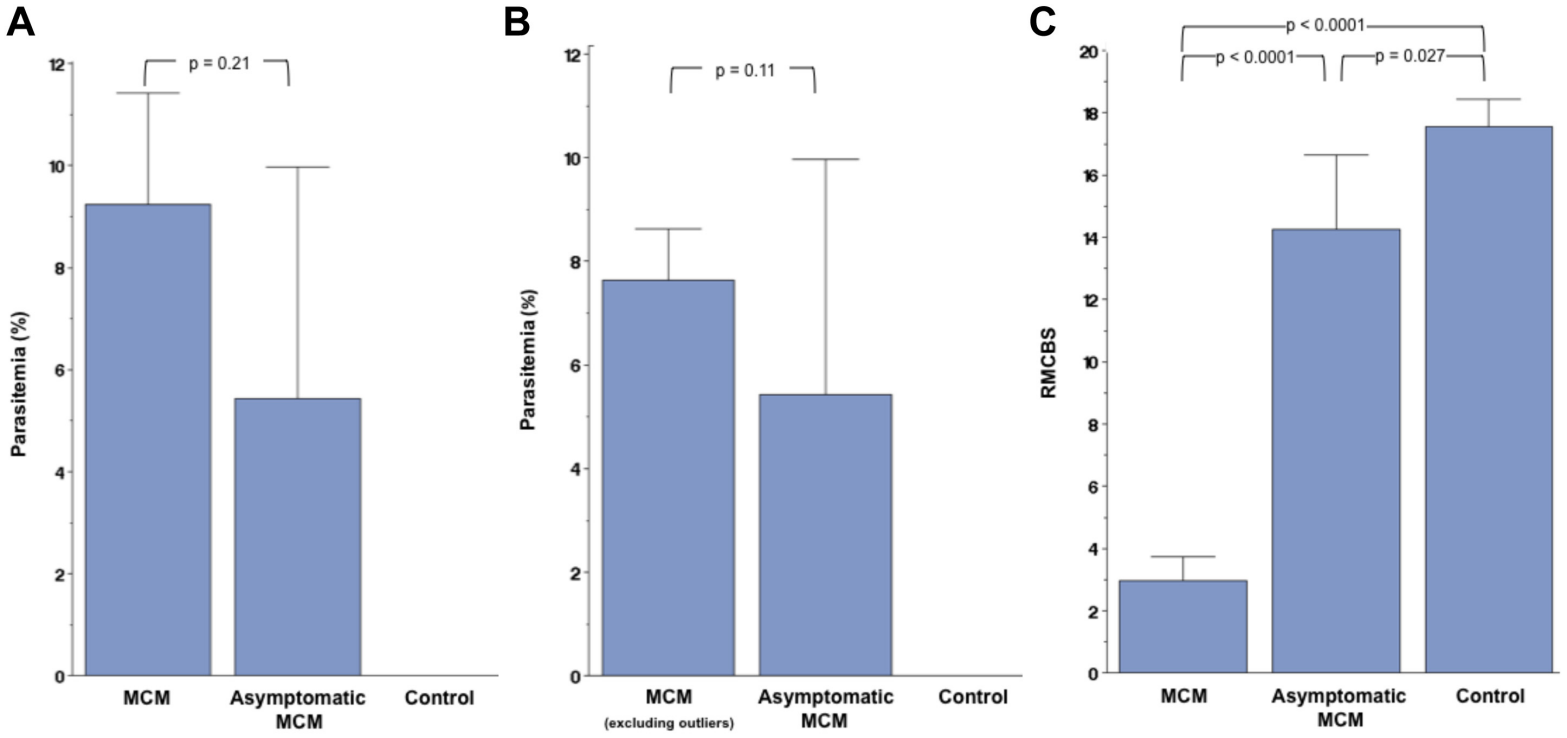
Overall outcome data. **A and B.** Compared cumulative parasitemia data from 5 experiments involving symptomatic MCM versus asymptomatic infected mice, p = 0.21 and 0.11, with (**A**) and without (**B**) outliers of parasitemia >20%, respectively. **C**. Cumulative RMCBS data from the same 5 experiments. Pair-wise comparison of symptomatic MCM mice versus control: mean difference −14.6 (95%CI −16.4 to −12.7, p<0.0001); the symptomatic MCM versus asymptomatic: mean difference −11.3 (95% CI −13.9 to −8.7, p<0.0001); and the asymptomatic MCM versus control mice: mean difference −3.3 (95% CI −6.3 to −0.3, p = 0.027). Tukey-Kramer's method was used to adjust for multiple comparisons.

Over the course of 5 experiments, we found that 36 of 40 (90%) of infected mice developed symptoms consistent with MCM between days 5 and 9 of infection, with a range of final RMCBS scores of 0 to 7 (mean 3.0), and a mean final parasitemia of 9.2% (range of 3.5%–37.3%; mean 7.6% without outliers greater than 20%, n = 3) (Figure 3). This is consistent with the prior literature on MCM outcomes [9], [10], [11]. The 4 asymptomatic mice (non-MCM) demonstrated a RMCBS range of 13–16 (mean 14.3) on the day of sacrifice (day 6–9) with a mean parasitemia of 5.4% (range 3.4%–9.6%). There was no significant difference in the parasitemia levels of the symptomatic and asymptomatic MCM mice (p = 0.21 and 0.11, with and without outliers of parasitemia >20%, respectively) (Figure 3A and 3B). Using Tukey-Kramer's method to adjust for multiple comparisons, the pair-wise assessments are as follows: comparing the final RMCBS of symptomatic MCM mice with control, the mean difference was −14.6 (95%CI −16.4 to −12.7, p<0.0001); the symptomatic MCM mice with asymptomatic MCM mice, the mean difference was −11.3 (95% CI −13.9 to −8.7, p<0.0001); and the asymptomatic MCM mice with the control mice, the mean difference was −3.3 (95% CI −6.3 to −0.3, p = 0.027) (Figure 3C). The utilization of the RMCBS allowed us to consistently and accurately follow the trajectory of the MCM disease process in each of these experiments, thus demonstrating reproducibility and speed.

**Figure 4:**
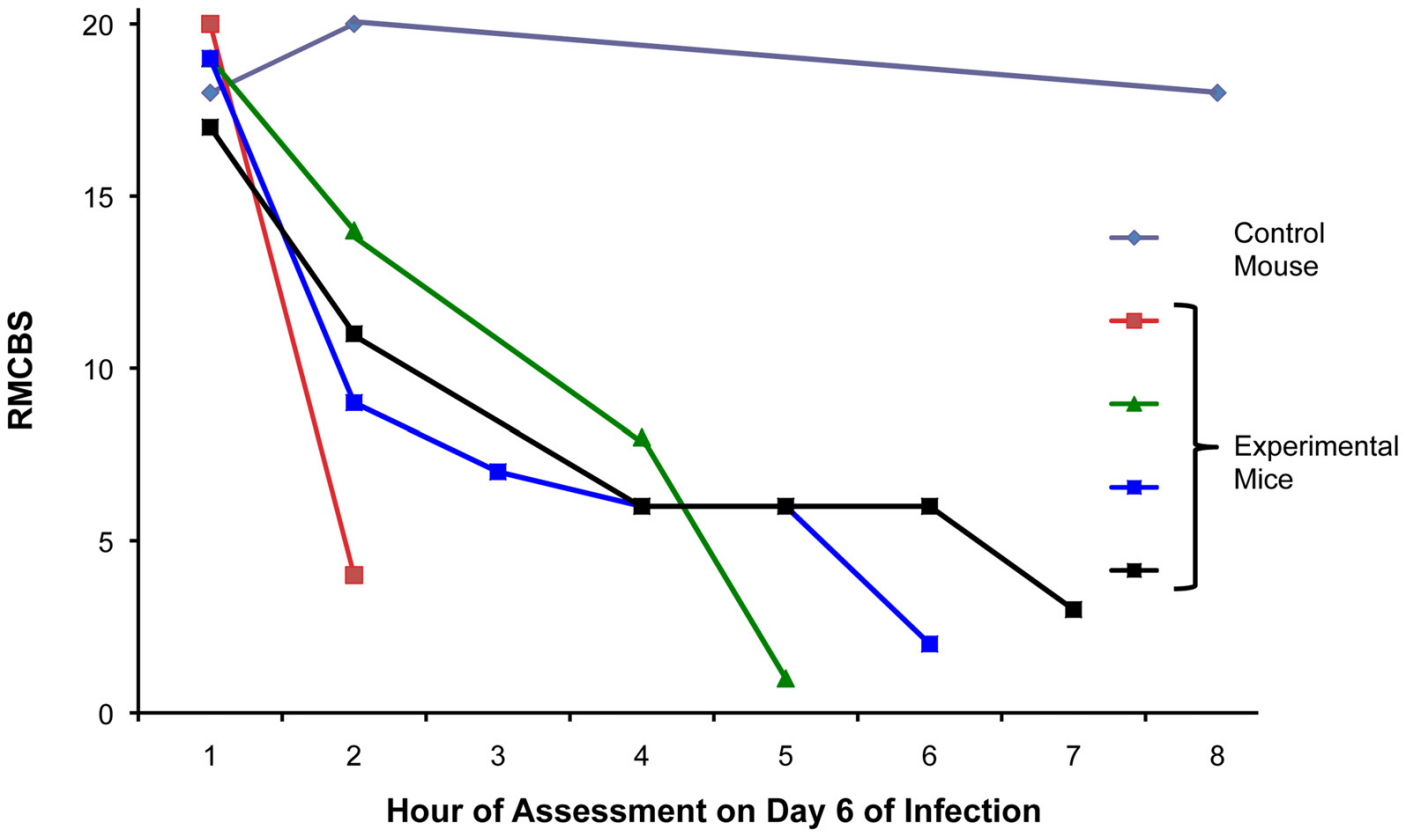
Increased frequency in surveillance via RMCBS. Mice were rapidly assessed at frequent intervals on day 6 of infection via the RMCBS, allowing the operator to closely follow the trajectory of disease progression.

Data from the previous experiments showed the greatest decline in the RMCBS between days 5 and 8 of infection (Figure 1). To determine more precisely the time course of clinical deterioration within this window, we next quantified the RMCBS at more frequent intervals (Figure 4). Twelve C57BL/6 mice with *P. berghei* ANKA infections and 3 non-infected control mice were followed daily via RMCBS and parasitemia. When the subjects began demonstrating symptoms, on day 6 of infection, we assessed the mice more frequently, as follows: if the RMCBS score was 16–20, mice were assessed every 12 hours; if the RMCBS score was 11–15, mice were assessed every 4 hours; if the RMCBS score was 6–10, mice were assessed every 2 hours; and if the RMCBS score was ≤5, the mice were considered moribund and were sacrificed. For clarity, we provide a representative figure including 4 experimental mice and one control mouse in Figure 4. We did not report these findings via mean RMCBS to demonstrate the mouse-to-mouse variability and individual disease curves.

**Figure 5:**
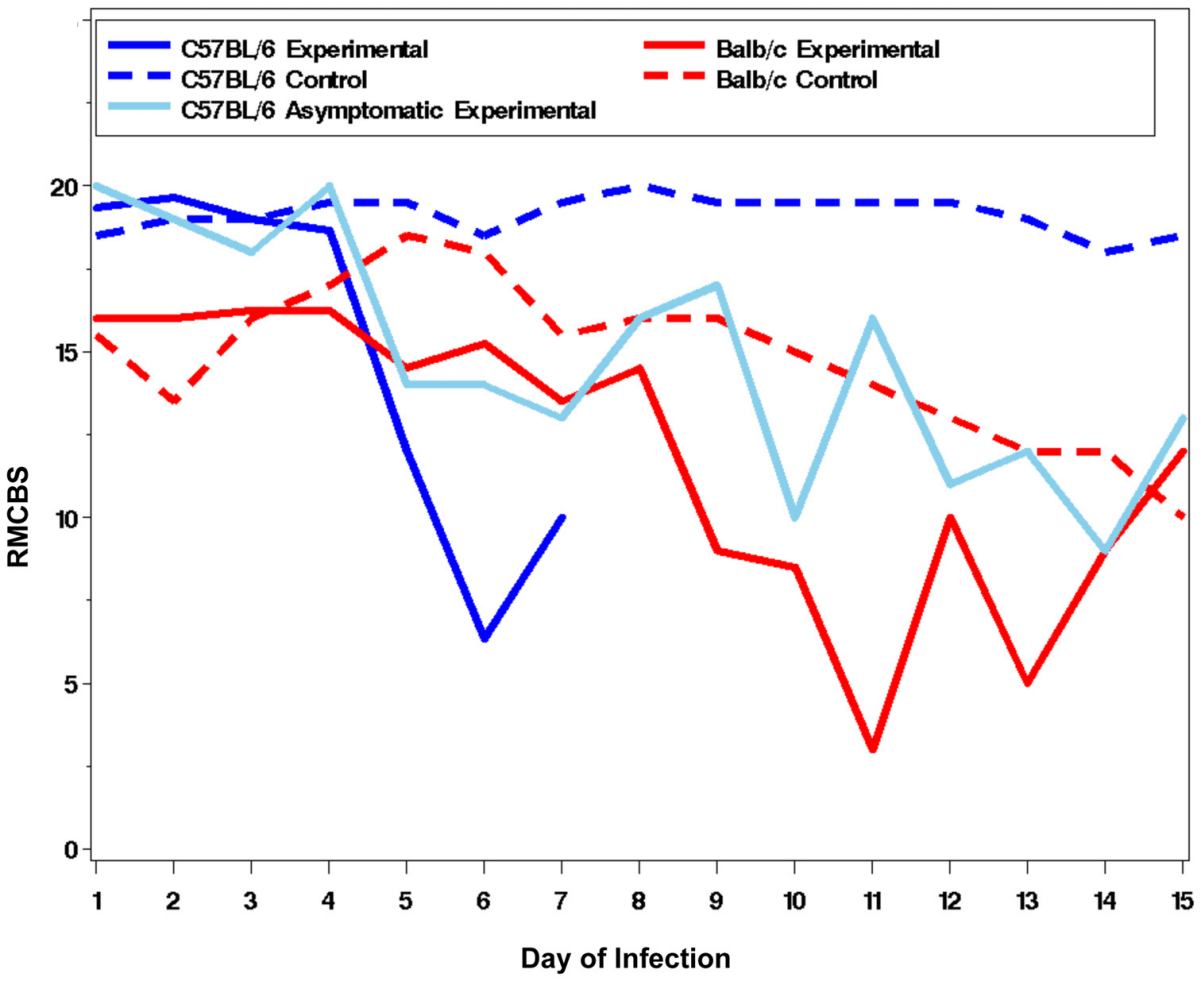
Variable RMCBS outcomes. Mean RMCBS curves for MCM-susceptible mice (C57BL/6, n = 3), an asymptomatic C57BL/6 mouse (n = 1), infected MCM-resistant mice (balb/c, n = 4), and non-infected control C57BL/6 (n = 2) and balb/c mice (n = 2). Comparing slopes of mean RMCBS curves: infected C57BL/6 versus infected balb/c (p = 0.01); infected C57BL/6 versus non-infected C57BL/6 controls (p = 0.004); symptomatic infected C57BL/6 versus asymptomatic infected C57BL/6 (p = 0.06, marginally significant); infected balb/c versus non-infected control balb/c (p = 0.48); and infected balb/c versus asymptomatic infected C57BL/6 and non-infected control C57BL/6 (p = 0.70).

**Figure 6:**
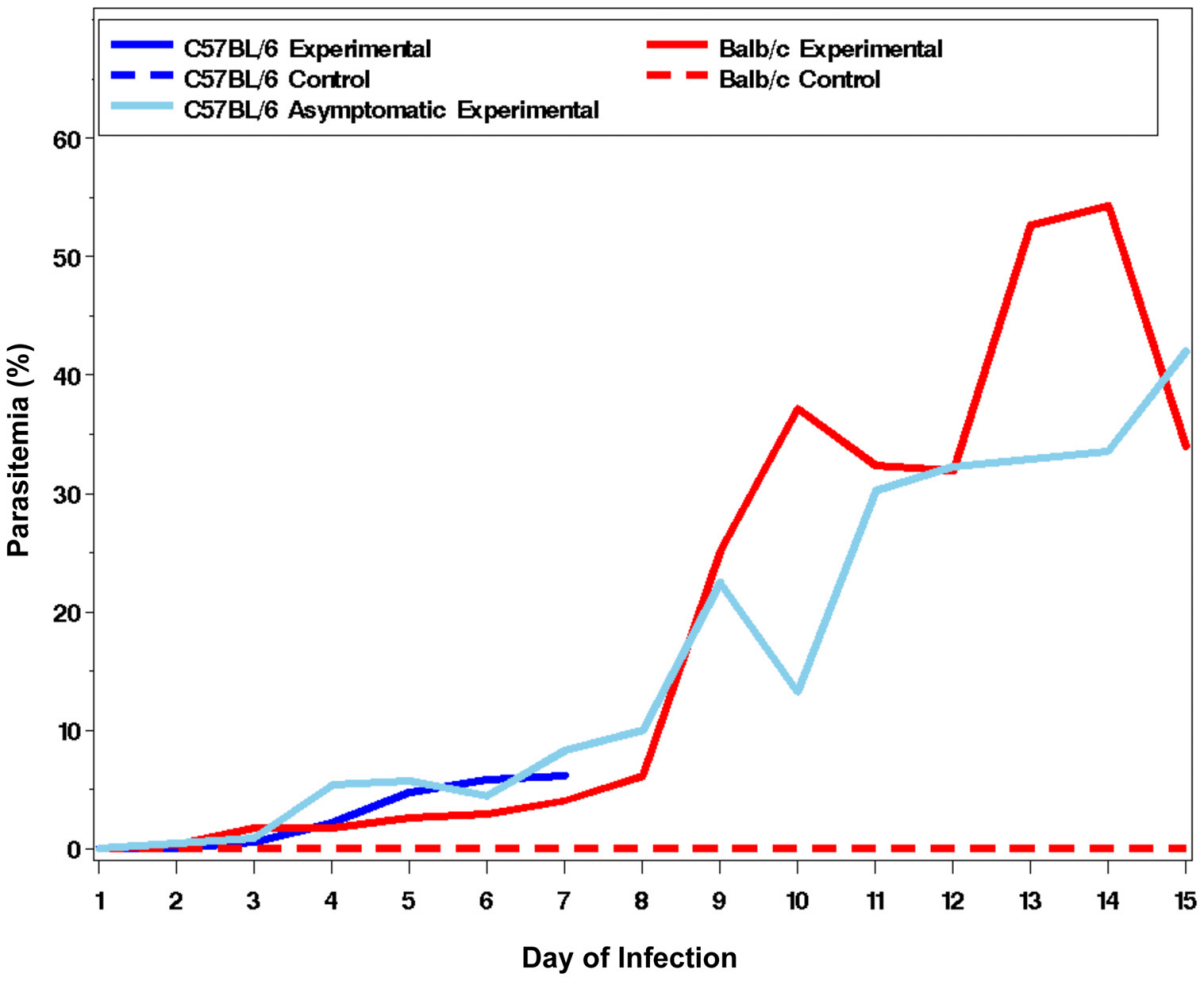
Parasitemia curves. Mean parasitemia curves for symptomatic MCM-susceptible mice (C57BL/6), MCM-resistant mice (balb/c), an asymptomatic MCM mouse (C57BL/6), and non-infected control mice (C57BL/6 and balb/c).

To further assess the value of the RMCBS to follow the trajectory of illness specific to the MCM model, we inoculated a known MCM-resistant mouse strain, balb/c, with PbA [12], [13], [14] and followed the disease progress via the RMCBS. In addition, we utilized the RMCBS to follow the C57BL/6 mice infected with PbA that did not develop symptoms of MCM, but rather survived over a week, developing high parasitemia. Figure 5 shows the mean RMCBS score for non-infected control C57BL/6 (n = 2, parasitemia 0%, intracerebral hemorrhages 0), symptomatic infected C57BL/6 mice (n = 3, peak parasitemia 6.2% on day 7, intracerebral hemorrhages 18–102), an asymptomatic infected C57BL/6 mouse (n = 1, peak parasitemia 42% on day 15, intracerebral hemorrhages 0), as well as non-infected control balb/c mice (n = 2, parasitemia 0%, intracerebral hemorrhages 0), and infected balb/c mice (n = 4, peak parasitemia 54.3% on day 14, intracerebral hemorrhages 0). There is a significant difference when comparing the mean RMCBS slopes of symptomatic infected C57BL/6 and infected balb/c (p = 0.01); a significant difference when comparing symptomatic infected C57BL/6 and non-infected C57BL/6 controls (p = 0.004); a marginally significant difference (p = 0.06) when comparing symptomatic infected C57BL/6 with asymptomatic infected C57BL/6; a non-significant difference when comparing infected balb/c and non-infected control balb/c (p = 0.48); and a non-significant difference (p = 0.70) when comparing infected balb/c with asymptomatic infected C57BL/6 and non-infected control C57BL/6. Figure 6 demonstrates the parasitemia curves for these same cohorts. Two experimental balb/c mice and one non-infected control balb/c mouse were sacrificed on day 7 to determine the presence of intracerebral hemorrhages. The RMCBS clearly delineates the rapid decline in the health of the experimental infected C57BL/6 mice. Note that the RMCBS follows the steady decline of the balb/c mice; however, the difference between the control and infected experimental balb/c mice is not as specific as in the C57BL/6 mice, making the RMCBS unsuitable for models utilizing the balb/c mice, and better suited for the MCM model.

### Correlating the RMCBS with histopathology

**Figure 7:**
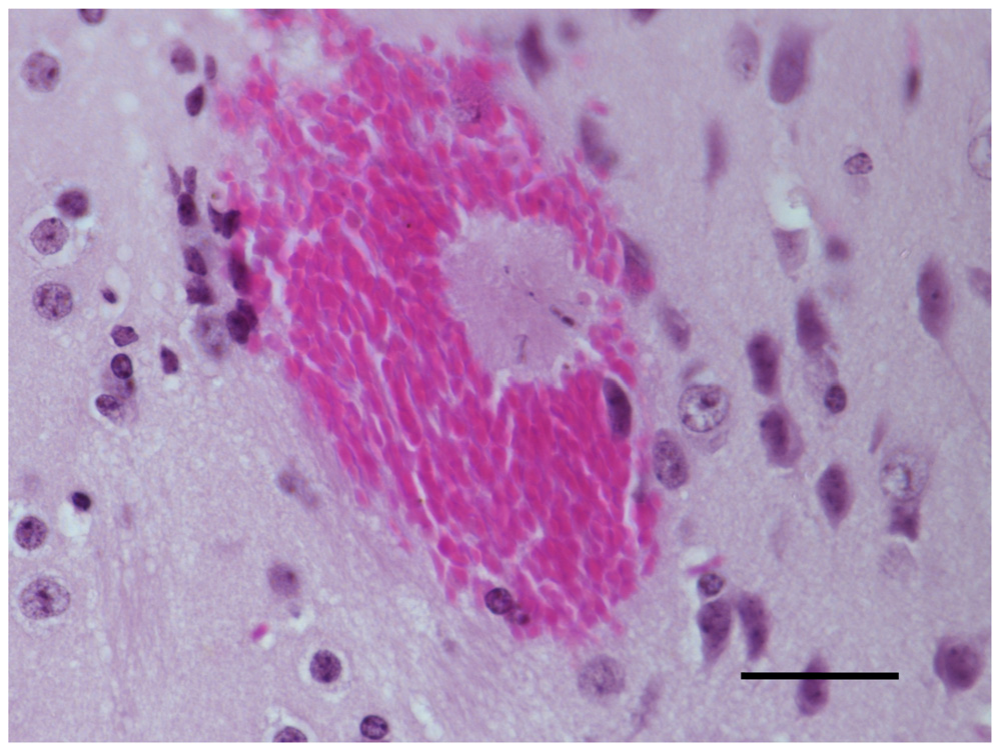
Intracerebral hemorrhage in a symptomatic mouse. PbA-infected mouse with final RMCBS of 5, H&E at 1000×, bar = 80-micrometers.

**Figure 8:**
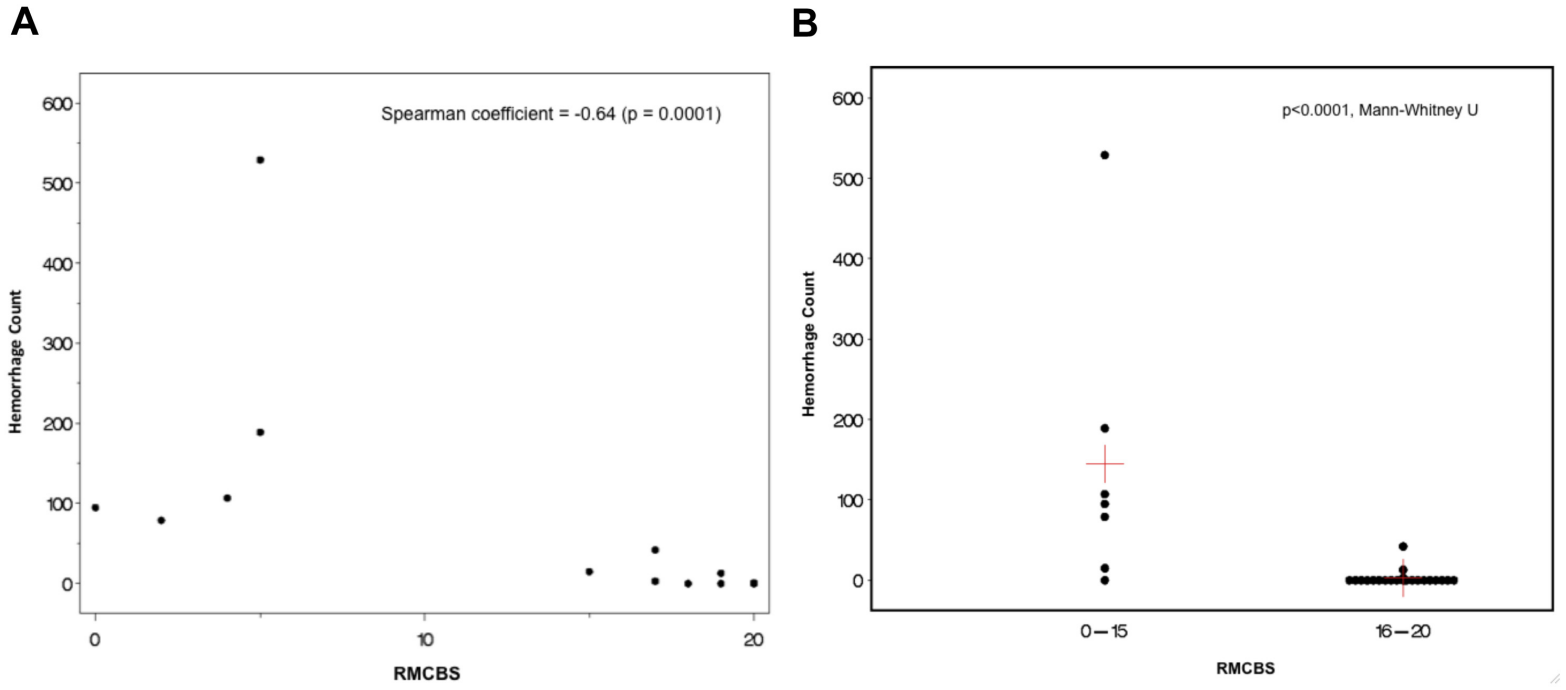
Correlation between RMCBS and number of hemorrhages. **A**. Mice were serially sacrificed on days 2, 4, 6, 8, 9 and 10, demonstrating a strong negative correlation between the number of intracerebral hemorrhages and the RMCBS score on the day of sacrifice, r = −0.64 (p = 0.0001, experimental n = 22, control n = 6). **B**. Comparing the total number of hemorrhages in cohorts of mice that scored between 16–20 versus 0–15 (p<0.0001, Mann-Whitney U). Red crosshairs = mean RMCBS.

Next, we investigated whether there was a correlation between the reduction in RMCBS and a concomitant increase in intracerebral hemorrhages, a known CNS pathology in MCM (See Figure 7). Hemorrhages were counted by an operator blinded to the clinical condition of the mouse, and were defined as a well-circumscribed collection of RBCs (10 to 100s) in the brain parenchyma, not associated with a blood vessel. The number of hemorrhages detected ranged from none (in non-infected control and infected asymptomatic mice) and 1 to 529 in symptomatic infected experimental mice. Figure 8A demonstrates a strong negative correlation between the number of hemorrhages and the RMCBS score on the day of sacrifice. Utilizing raw values of the RMCBS, the Spearman Correlation coefficient between hemorrhagic counts and RMCBS was −0.64, (p = 0.0001). While significant, this Spearman Correlation may only be −0.64 because the number of hemorrhages is only one metric. It stands to reason that the greater the number of hemorrhages, the greater likelihood the subject will develop pathology. However, a single hemorrhage located in an area vital to central functions, namely respiratory drive (i.e., brainstem and the medulla oblongata), can lead to terminal respiratory failure. It is likely that the location of the hemorrhages is just as important as the total number.

**Figure 9:**
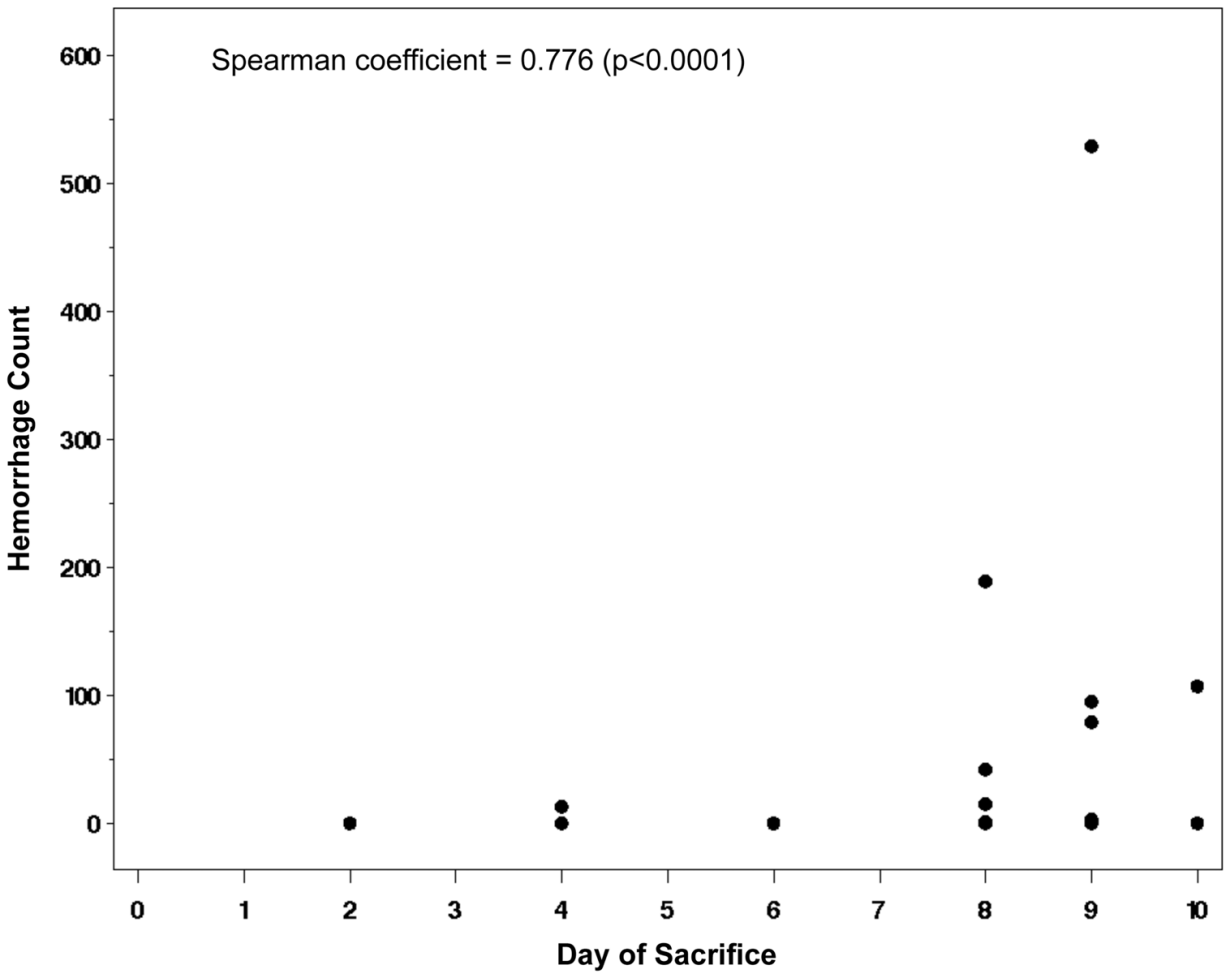
Hemorrhages and day of sacrifice. Intracerebral hemorrhage tally in experimental mice versus day at which subject was sacrificed, demonstrating a positive correlation. Spearman Correlation = 0.776, p<0.0001.

When mice are dichotomized according to the RMCBS, mice achieving a RMCBS of 0–15 (n = 6) demonstrate a total hemorrhage count of 1014, versus the cohort achieving a RMCBS of 16–20 (total n = 22 minus 6 control = 16 experimental) with a total hemorrhage count of 59 (p<0.0001, Mann-Whitney U) (Figure 8B). To reinforce that infected subject animals will develop an increasing number of hemorrhages as the infection progresses, we demonstrate a positive correlation between the day of sacrifice and the number of hemorrhages (Spearman Correlation = 0.776, p<0.0001, not including control mice, which were expected to lack hemorrhages regardless of the day sacrificed) (Figure 9).

Detectable parasitemia levels on the day of sacrifice ranged from 0.1% to 7.1%. Note that mice with documented infections, while demonstrating a high RMCBS (>15) still demonstrated intracerebral hemorrhages. In fact, one experimental mouse sacrificed early in the experiment (day 4) demonstrated intracerebral hemorrhages, but had not yet demonstrated the clinical phenotype, nor did it have a detectable level of parasitemia. In brains taken from mice with low RMCBS (≤12), hemorrhages were seen in all areas of the brain, representing Bregma +5 through −9. Anatomical locations in which hemorrhages were consistently found include: olfactory bulb, motor cortex, medulla, pons, brainstem, and the cerebellum. Data from two experimental mice were not included because one did not develop infection, and a second was found dead and the brain tissue was uninterpretable.

Only one experimental mouse was sacrificed on day 10 (RMCBS 4, parasitemia 4.9%, hemorrhage count 107). The other mice intended to be sacrificed in this cohort all became moribund on earlier days (8 and 9) and were sacrificed with those respective cohorts. Of note, the parasitemia for this experiment was slow to rise: 0%–0.1% by day 5, and 0%–1.6% by day 6. It can be argued that there is a cascade of cellular-molecular pathophysiology that is triggered by a threshold level of parasitemia. This may be a reason why clinical pathology, histopathology, and lower RMCBS scores were not seen until days 8, 9, and 10. This variable in the MCM model is another reason why the RMCBS should be used to assess the level of disease. Empirically introducing an adjuvant therapy on day 5 of infection may lead to falsely reassuring outcomes because the parasitemia and cellular-molecular pathophysiology have yet to fully develop.

### Evaluation of an antimalarial drug using the RMCBS as a guide for treatment

**Figure 10:**
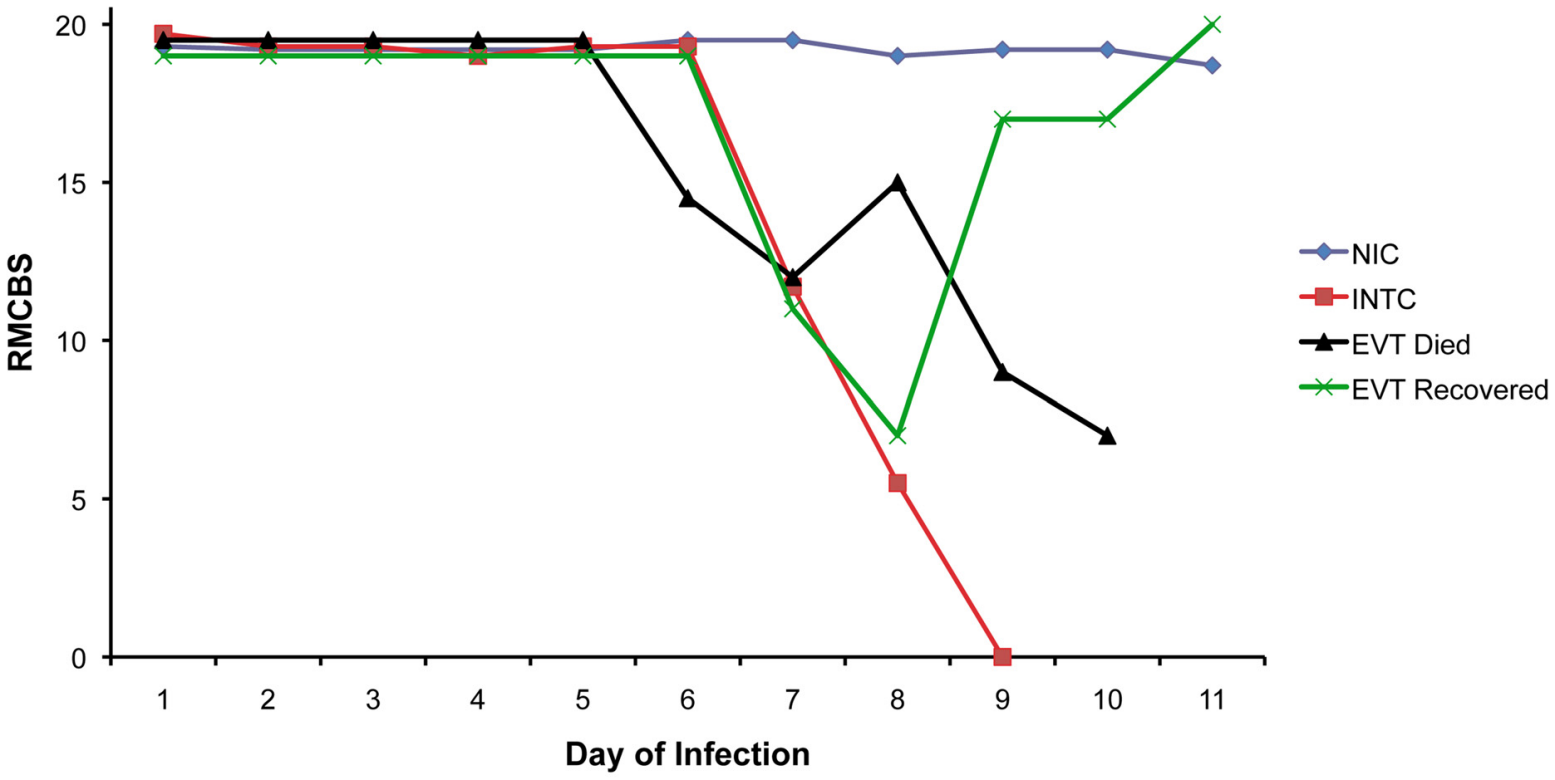
Outcome of chloroquine treatment of symptomatic mice with RMCBS≤12. Mean RMCBS scores of Non-Infected Controls (NIC, n = 6); Experimental Viable Treated (EVT, n = 5, peak parasitemia range 4.2%–5.9%, hemorrhage range 23–108 [mean 51]); Infected Non-Treated Controls (INTC, n = 15, peak parasitemia range 1.9%–12.1%, hemorrhage range 1–243 [mean 42]). An EVT animal received treatment on day 7 of infection (at RMCBS of 11), clinically worsened (RMCBS 7 on day 8), but made a full recovery.

In a preliminary experiment on the utilization of the model, we examined the effectiveness of the RMCBS in chloroquine treated mice (Figure 10). To do this we had to select a RMCBS score at which to initiate treatment. In prior experiments, none of the control mice developed a score less than 15 (see Figure 1). In addition, any infected mouse that developed a RMCBS score of 12, or less, became moribund within 24–48 hours; none spontaneously recovered from a disease state with such a low score. Therefore we chose a conservative cut-off score of 12 to label mice with MCM, providing a threshold for treatment. Thus symptomatic animals that developed a RMCBS score of 5–12, within the experimental period of 5–9 days were eligible for treatment. Mice that presented with a score of 5, or less, were euthanized.

Categories of mice involved in this experiment are summarized as follows: Non-Infected Controls (NIC, n = 6); Experimental Viable Treated (EVT, n = 5); Infected Non-Treated Controls (INTC, n = 15). EVT and INTC mice (total n = 20) were inoculated with PbA as described above, 15 of which demonstrated a RMCBS score consistent with a moribund state, were thereby labeled INTC, left untreated, and were euthanized. Five mice with RMCBS≤12, who were not moribund, were treated with chloroquine (thus labeled EVT). Four mice of the EVT group ultimately succumbed, having been treated on days 6, 6, 8, and 9, with RMCBS scores of 9, 10, 11, and 11, respectively. All of these mice developed a RMCBS of 5, or lower, the day following the initiation of treatment and were euthanized or died. The fifth EVT mouse was treated on day 7, at a RMCBS score of 11, demonstrating a RMCBS nadir of 7 the following day, ultimately making a full recovery. This mouse also developed complete resolution of its parasitemia (peak parasitemia on the initial day of treatment was 5.2%). Overall, this represents a mortality rate of 80% in the EVT group, despite treatment. In contrast, 100% of the INTC mice died, or were euthanized when found moribund. The data in Figure 10 shows the trajectory of the mean RMCBS between these different groups. For clarity, four mice that were intended for the INTC and EVT groups that did not develop infection (n = 2) or were infected but asymptomatic (n = 2) were not included in these results. The RMCBS, however, did accurately reflect the disease state of these animals: high normal (≥19) for uninfected mice, and low normal (≥14) for infected asymptomatic mice.

**Figure 11:**
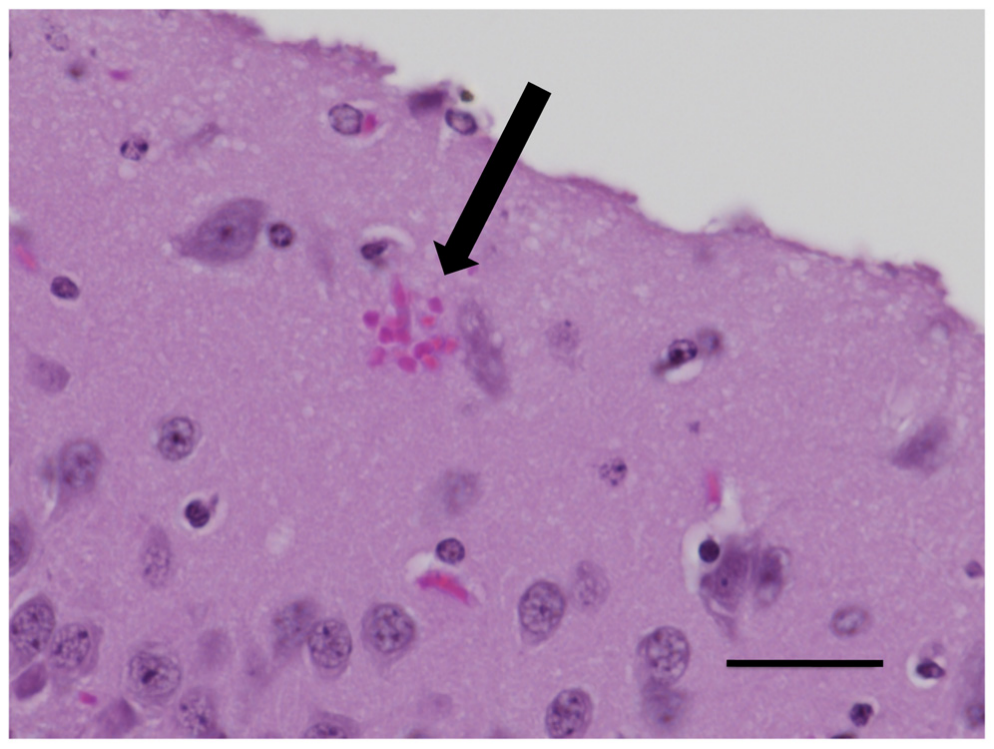
Hemorrhage in recovered EVT mouse. Only three small intracerebral hemorrhages were found on post-mortem examination of the EVT mouse that recovered (Figure 10), one of which is demonstrated here (black arrow). H&E, 1000×, bar = 80-micrometers.

All animals in the EVT and INTC groups demonstrated intracerebral hemorrhages, assessed as described above, using 6 H&E-stained sagittal sections per mouse. The mice from the INTC group demonstrated a range of 1–243 hemorrhages (mean 42, peak parasitemia range of 1.9%–12.1%). This hemorrhage count does not include 3 INTC mice whose brain tissues were considered uninterpretable. The mice from the EVT group demonstrated a range of 23–108 hemorrhages (mean 51, peak parasitemia range of 4.2%–5.9%). Of note, the animal that survived in the EVT group (peak parasitemia 5.2%) was treated at an RMCBS of 11, and demonstrated only 3 small intracerebral hemorrhages on post-mortem inspection (Figure 11). These data further demonstrate that the score is closely associated with histological evidence of damage. This suggests that there may be a threshold in the number of hemorrhages that leads to a rapid decline in the subject's health, pushing the subject to become moribund. This further reinforces that an adjuvant therapy is needed to help reduce the mortality of CM. The RMCBS may facilitate studies designed to utilize disease trajectory to help identify the optimal window for treatment.

## Discussion

Until a safe and effective adjuvant therapy is discovered, CM will remain the most virulent and deadliest clinical manifestation of malaria. Twenty-percent of treated patients with CM succumb, and most die within 24–48 hours. Human patients with CM often come to medical attention well into coma, reflecting both the rapidity by which CM is manifest as well as the socio-economic challenges faced by the populations living in malaria-endemic areas. By this time, a cascade of incompletely understood pathological events is well underway. Adjuvant therapy trials in humans aimed at halting these processes have not demonstrated promising results [15]. More specifically, mannitol, steroids, antipyretics, anti-seizure control, pentoxyfylline, and defuroxamine, among others, have been proposed, used in limited instances, or been shown to be detrimental, as in the case of steroids and defuroxamine (Reviewed by Mohanty et al. [16], and Mturi et al. [17]). An adjuvant therapy remains elusive.

Murine models of CM have been widely used to further elucidate the pathophysiology of CM, as well as reveal potential adjuvant therapies. To begin, major similarities and differences between the MCM model and human CM have been described. On a clinical basis, both MCM and human CM share phenotypes of CNS dysfunction, including hemiparesis, seizures, coma, and a rapid decline in health after the onset of illness (reviewed by Hunt et al.) [18]. On the basis of histopathology, both share intracerebral hemorrhages, histological signs of blood-brain barrier dysfunction, and WBC sequestration [19], [20], [21]. Conversely, the finding of sequestered parasitized-RBC (pRBC) in the cerebral vasculature remains consistent in human CM [22], whereas this phenomenon is inconsistently reported in the MCM model [18], [23], [24], [25].

In light of these discrepancies, some authors have recently questioned the merit of the MCM model [15]. However, multiple authors contend that the MCM model, albeit imperfect, can be used to enhance the study of human CM [26], [27], [28], [29]. There is a clear call for a MCM model that better emulates the human CM condition.

A rapid murine assessment scale, as described in this study, is imperative to emulating the human CM manifestation, while providing operators with the ability to accurately label mice as symptomatic, with a high degree of confidence of underlying pathology, consequently providing a window during which rescue and neuroprotective adjuvant therapies can be assessed.

Most MCM studies involve a binary evaluation of CM: the subject is labeled affected, or not, using traditional descriptive criteria [16], [30], [31], [32]. A more objective neurological assessment score, the SHIRPA protocol, has been developed for rodent models [33], [34], [35], [36], and has been applied to the MCM [9], [10], [11], [37], [38], involving a maximum of 12 to 18 animals per experiment [9], [10], [11]. It requires 40 parameters of observation, and a complete assessment can take up to 20–30 minutes per mouse, making this score time-consuming. Despite original projections [33], this decreases the level of throughput and could be considered suboptimal for frequently assessing a large number of subjects and utilizing a threshold for treatment. We contend that using this protracted score with larger numbers of animals in a single cohort (n of 50 to 100) would prove difficult, especially when introducing interventions during the short symptomatic period (∼24h) prior to death. Furthermore, aspects of the SHIRPA score highlight processes in the mouse that are not directly attributable to CNS dysfunction (i.e., defecation and urination frequency). In an effort to focus the SHIRPA score, recent work by Martins et al. [37] combines the SHIRPA score and logistic regression modeling to predict the likelihood that a murine subject will develop fatal CM. Of the 23 SHIRPA parameters with the highest predictive yield that were assessed on days 5 and 6 of infection, all 10 corresponding parameters of the RMCBS are included. In parallel to the work completed by Martins et al., we independently developed the RMCBS as a tool to be used to quantitatively recognize the development of murine CM in real time, validating its use in adjuvant therapy trials.

To date, most trialed therapies have not been initiated after the onset of MCM symptoms, making the conclusions less impactful. Many authors have initiated empiric adjuvant therapy prior to, on, or just after the first day of inoculation [11], [39], [40]. Alternatively, empiric treatments have been provided around the proposed time MCM symptoms have historically been documented to start, on days 3 to 7, post-infection [30], [39], [41], [42]. While the results of these experiments have been promising, they do not fully emulate the human clinical experience—patients are treated at the onset of clinical symptoms, and often, much later. A murine-based model of CM that better emulates the human CM experience is needed, and it must include a neurobehavioral score that is rapidly deployed.

We have developed the RMCBS to enhance the MCM model, allowing the model to more closely emulate human CM. The RMCBS allows an operator to quickly and objectively assess multiple mice during the period in which the mice demonstrate a rapid decline in health. The RMCBS can be used to establish the time at which pharmacotherapy should be given, detect a response to the therapy, correlate the clinical course with quantifiable intracerebral histopathology, and ultimately trace the path to recovery, making this score ideal for drug discovery for CM.

Because many patients with CM present to the hospital already in coma, an adjuvant therapy that halts the pathological process is desperately needed. In addition, at present, there is no way to predict which malaria patient will plummet into CM, and which will not. Perhaps a biomarker that reflects a patient's risk of developing CM can help guide prophylactic treatment. In parallel, an adjuvant neuroprotective therapy is needed to curb the number of children with long-term effects of CM. The RMCBS can be used to guide further research endeavors aimed at discovering these vital pieces of information.

## Materials and Methods

### Ethics Statement

All procedures were approved by the Northwestern University and the University of Notre Dame IACUC committees.

### Description of the RMCBS

The RMCBS consists of 10 parameters, and each parameter is scored 0 to 2, with a 0 score correlating with the lowest function and a 2 score, the highest. An animal can achieve an accumulative score of 0 to 20. During the first 90 seconds of assessment, the mouse is placed in the top left corner of an observation box (length, 31.8 cm [12.5 in]; width, 19.8 cm [7.8 in]; height, 10.5 cm [4.1 in]) with a grid floor and is assessed for hygiene-related behavior, gait, body position, exploratory behavior, and balance. In the subsequent 90 seconds, the mouse is assessed for reflexes, limb strength, and self-preservation actions. The tail blood sampling for parasitemia is incorporated into the score assessment. An illustrative table (Table 1) is provided for more specific descriptions of the assessments. Furthermore, a video recording of the score, useful for training, is available in the on-line Supporting Information section (see Video S1 [low-resolution, 10.3MB]/Video S2 [high-resolution, 336MB]).

### The murine model and RMCBS

The parasite strain *Plasmodium berghei* ANKA used in these studies was supplied by MR4 (uncloned line MRA-311, depositor Thomas McCutchan). A known CM-susceptible mouse strain was used: 4–6 week old female C57BL/6 mice (C57BL/6NCRL from Charles River Breeding Laboratories, Portage, Michigan, USA), which were acclimated in the Research Resource Center of Northwestern University for 2 weeks, per protocol, with food and water *ad libitum*. Any mouse with obvious physical deformities or size discrepancies was not included in the experiment. When the mice reached 6–8 weeks of life, 16 experimental mice were inoculated intraperitoneally (IP) with 1×10^6^ PbA (0.1mL) taken from a homologue donor, which had been infected from frozen stock. Four control mice were injected IP with 0.1mL of sterile PBS. Each cage contained four experimental mice with one control mouse. One control mouse was discarded due to congenital growth retardation, leaving three control mice. Mice were assessed via the RMCBS every 24 hours. Attempts were made to assess the mice at the same time each day, usually the evening hours, for every experiment; however, this was not always achieved, and mice needed to be assessed frequently, at variable times of the day. Blood was sampled daily from tail tip phlebotomy, and parasitemia was determined by tabulating the number of Giemsa-stained parasitized-RBCs out of a total of 500 RBCs, counted by a blinded operator using 40× light microscopy. Early experiments revealed that a mouse with a RMCBS of 5, or less, would die within 1–4 hours, and such mice were labeled moribund and were anesthetized with 10% pentobarbital IP. Mice were exsanguinated via heart puncture and/or inferior vena cava sever, and the brains were carefully excised and placed in pH-balanced 10% formalin for 1–2 weeks.

We further assessed the effectiveness and specificity of the RMCBS by comparing the trajectories of illness in subjects of a non-MCM model and symptomatic and asymptomatic MCM mice. We examined the RMCBS in PbA-infected C57BL/6 (known MCM-susceptible) mice and PbA-infected balb/c (known MCM-resistant) mice. Experimental and control mice were inoculated, assessed, and euthanized as described above. Intracerebral hemorrhages were evaluated by an operator blinded to the subject phenotype, viewing 5 H&E-stained sagittal sections under 40×.

### Brain histopathology and analyses

Brains were cut along the mid-sagittal line and the separated hemispheres were positioned so that the newly cut surface was presented, open-faced, to the microtome. The brains were then fixed in paraffin blocks and 5-micrometer sections were cut along the sagittal plane (interaural), so that both hemispheres were cut side-by-side along the same anatomic plane and the juxtaposed sections were placed on a glass slide. We obtained 5 such sections per mouse, each separated by 40 micrometers, thus producing 10 sections per brain. Because the locations of predominant pathology in MCM have yet to be fully delineated, we cut the brain along the sagittal plane, thereby including all of the Bregma positions, allowing the operator to visualize the entire brain structure. Sections were stained with H&E and visualized with light microscopy.

### Correlating the RMCBS with histopathology

Twenty-four experimental mice were inoculated with PbA, and 6 control mice were injected with sterile PBS, and were assessed daily, as described above. Cohorts of infected (n = 4) and uninfected mice (n = 1) were sacrificed on days 2, 4, 6, 8, 9, and 10 after infection. Mice were euthanized and brains were excised and sectioned as described above, with the following variation: after euthanization and exsanguination, the head was removed at the base of the cervical spine, the skin and fur removed, and the whole head was placed in 10% formalin for 2 weeks. At that time, after the tissue was well fixed, the brains were carefully excised and placed in 10% formalin for 2 additional weeks. The brains were then sectioned as described above. This resulted in 10 H&E-stained sections, 5 of each hemisphere, for each mouse, which were subsequently masked and coded, blinding the operator to the clinical phenotype of the source mouse. The total number of hemorrhages was tabulated for all ten sections per mouse. A hemorrhage was defined as a clearly circumscribed collection of blood within the brain parenchyma that was not a part of the vascular system or part of an area of obvious trauma caused by instrumentation. See Figure 7 for an example of a hemorrhage seen in a symptomatic mouse.

### Evaluation of an antimalarial drug using the RMCBS as a guide for treatment

Twenty-four C57BL/6 mice were injected with PbA, as described above, and when a mouse reached a score between 12 and 5, it was IP-injected with 0.3 mg of chloroquine IP, based on an assumed 30-gram weight. These mice were treated daily with IP chloroquine, receiving daily RMCBS and parasitemia assessments. Mice were sacrificed when they became moribund (RMCBS≤5), or at the end of the experiment, on day 11. Non-infected and infected control mice were inoculated and assessed daily as described earlier, but were not treated with chloroquine. The non-infected control mice were sacrificed at the end of the experiment, on day 11. Brain handling, tissue staining, and parasitemia assessment were carried out as described above.

### Statistical Methods

To investigate the relationship between RMCBS and the day of infection, we fit a random intercept random slope mixed model with the RMCBS score as the outcome, and day of infection and group assignment (i.e., experimental versus control) as covariates. In order to increase the model fit to accommodate trends in the two groups, both linear and quadratic terms for day of infection are included in the model. Additionally, we limited the values of RMCBS scores to be non-negative.

Comparisons of RMCBS were also assessed at day 8 between the control and the experimental group with *t*-test for Figure 1. Finally, we compared the difference in the number of total hemorrhages in the two groups of RMCBS scores (0–15 versus 16–20) using the Mann-Whitney U analysis (Figure 8B). All statistical analyses were carried out using SAS/STAT software, Version 9.2 of the SAS system for UNIX, and the results were graphed using SAS/GRAPH (SAS Institute, Cary, NC).

## Supporting Information

Video S1 RMCBS Training Video. This training video covers the 10 parameters used in the RMCBS. Video and audio are used to demonstrate each parameter. A full scoring session is demonstrated for a single subject, as well. A higher resolution video file (336MB) can be downloaded (Video S2), or requested from corresponding author RWC.
(10.55 MB MOV)

Video S2 High-resolution RMCBS Training Video. Higher resolution version of Video S1. 336MB video file.
(343.25 MB MOV)
